# Two studies in one: A propensity-score-matched comparison of fingolimod versus interferons and glatiramer acetate using real-world data from the independent German studies, PANGAEA and PEARL

**DOI:** 10.1371/journal.pone.0173353

**Published:** 2017-05-05

**Authors:** Jonathan Alsop, Jennie Medin, Christian Cornelissen, Stefan Viktor Vormfelde, Tjalf Ziemssen

**Affiliations:** 1 Numerus Ltd, Wokingham, United Kingdom; 2 Novartis Pharma AG, Basel, Switzerland; 3 Novartis Pharma GmbH, Nuremberg, Germany; 4 Center of Clinical Neuroscience, Carl Gustav Carus University Clinic, Dresden University of Technology, Dresden, Germany; University of Oxford, UNITED KINGDOM

## Abstract

**Background:**

This study compared outcomes following fingolimod or BRACE treatments (beta-interferons/glatiramer acetate) in patients with active MS (≥ 1 relapse in the previous year) following previous BRACE treatment.

**Methods and findings:**

Patients with active MS who previously received BRACE were identified from German prospective, observational studies, PANGAEA and PEARL. A novel methodology was developed to compare outcomes between propensity-score-matched cohorts (3:1 ratio) from the independent single-arm studies. Patients in PANGAEA (n = 1287) experienced 48% fewer relapses per year than those in PEARL (n = 429; annualized relapse rate ratio: 0.52; *p* < 0.001). The risk of 3-month or 6-month confirmed disability progression (CDP) was reduced in PANGAEA versus PEARL (3-month: 37% reduction; hazard ratio [HR], 0.63; *p* < 0.001; 6-month: 47% reduction; HR, 0.53; *p* < 0.001). A higher proportion of patients in PANGAEA (n = 1234) than PEARL (n = 401) were free from relapses and 3-month (65.7% vs 38.7%; *p* < 0.001) or 6-month (68.2% vs 39.2%; *p* < 0.001) CDP. The probability of confirmed disability improvement was higher in PANGAEA (n = 1163) than PEARL (n = 372; 3-month: 175% increase; HR, 2.75; *p* < 0.001; 6-month: 126% increase; HR, 2.26; *p* < 0.001). Patients in PANGAEA (n = 149) were less likely than those in PEARL (n = 307) to have taken sick leave (proportion with 0 days off work: 62.4% vs 44.6%; *p* = 0.0005). For change in disease severity from baseline (assessed by clinicians using the Clinical Global Impressions scale; PANGAEA, n = 1207; PEARL, n = 427), a larger proportion of patients had subjective improvement and a smaller proportion had worsening status in PANGAEA than PEARL (improvement: 28.2% vs 15.2%; worsening: 16.4% vs 30.4%; *p* < 0.0001).

**Conclusions:**

Fingolimod appears to be more effective than BRACE in improving clinical and physician-/patient-reported outcomes in individuals with active MS.

## Introduction

Multiple sclerosis (MS) is a chronic, inflammatory, degenerative disease of the central nervous system [1]. It is a leading cause of disability in young and middle-aged people, and affects approximately 2.3 million individuals worldwide [2]. Most patients (80–85%) present with the relapsing–remitting form of MS (RRMS), which is characterized by clearly defined symptomatic attacks (relapses) followed by periods of remission [3]. Loss of function in RRMS is caused by two types of damage. Widespread diffuse damage starts early in the disease and often goes unnoticed [4–7]. It is associated with progressive reduction in brain volume and accumulated loss of physical and cognitive function [4–7]; brain volume loss appears to be an important predictor of future disability [7]. The other type of damage is focal damage (distinct inflammatory lesions detected by magnetic resonance imaging [MRI]). Focal damage is associated with relapses, which are primary clinical manifestations of RRMS, that are followed by stretches of full or partial recovery [1, 5]. Incomplete recovery from relapses can lead to an accumulation of disability in patients with RRMS [1]. Patients with this form of MS initially experience relapses at a mean frequency of one per year, with wide inter-individual variation [1]. The frequency of relapses typically decreases over time [1].

Treatments for MS traditionally aim to modify the disease by reducing the number of relapses and delaying the progression of disability [8]. Glatiramer acetate (GA) and beta-interferons (IFN beta) collectively comprise the BRACE treatments (Betaseron^®^, Rebif^®^, Avonex^®^, Copaxone^®^, Extavia^®^), which are widely approved as disease-modifying therapies (DMTs) for MS. For many patients, however, these injectable therapies have suboptimal tolerability profiles and are only partially effective [8, 9]. Approximately one-quarter of patients with RRMS receiving BRACE therapies continue to experience relapse activity (estimates range from 18% to 46% for IFN beta [10–14] and from 18% to 50% for GA [14–17]). These patients with active MS usually continue to experience breakthrough disease unless their treatment is switched to an alternative agent with greater efficacy/effectiveness [14, 18].

Fingolimod, the first oral therapy approved for MS, was licensed in the EU in 2011 for the treatment of patients with highly active RRMS, which includes those who have high disease activity despite previously receiving at least one DMT [19]. Patients in this clinically relevant population may have received a BRACE therapy, the oral therapies dimethyl fumarate or teriflunomide, or the second-line infusible therapy natalizumab [19], which is associated with safety concerns owing to an increased risk of progressive multifocal leukoencephalopathy during long-term use [20, 21]. Fingolimod has been shown to reduce both focal and diffuse damage in patients with RRMS [22–24]. In three phase 3 trials, fingolimod was found to have greater efficacy in reducing the number of relapses, slowing disability progression and improving MRI and brain atrophy outcomes compared with placebo (FTY720 Research Evaluating Effects of Daily Oral Therapy in Multiple Sclerosis [FREEDOMS] and FREEDOMS II) or intramuscular IFN beta-1a (Trial Assessing Injectable Interferon vs FTY720 Oral in Relapsing–remitting Multiple Sclerosis [TRANSFORMS]) [22–24]. *Post hoc* analyses of data from FREEDOMS and FREEDOMS II have demonstrated the efficacy of fingolimod versus placebo in subgroups of patients with high disease activity despite previous DMT use [25, 26]. Similarly, a *post hoc* analysis of data from TRANSFORMS found a significant reduction in relapses for fingolimod versus IFN beta-1a in patients with high disease activity despite DMT use in the year before the study [27].

Data from phase 3 trials of fingolimod have been complemented by real-world evidence collected from administrative claims databases and registry studies [28]. Administrative claims database studies have shown that fingolimod was associated with significantly lower relapse rates than BRACE therapies in patients with MS whose treatment was switched from IFN beta 1a or 1b [29] and in those with a history of relapse [30]. The relative effectiveness of switching to fingolimod or BRACE therapies following disease activity (relapse or disability progression) has also been investigated in a global MSBase registry study [31]. After switching, the fingolimod group had a significantly lower mean relapse rate (0.31 vs 0.42; *p* = 0.009) and risk of disability progression (hazard ratio [HR], 0.53; 95% confidence interval [CI]: 0.31–0.91; *p* = 0.02) than the propensity-score-matched BRACE cohort [31].

To investigate prospectively the long-term safety and efficacy of fingolimod and BRACE therapies further in daily clinical practice in a large cohort of patients with active RRMS, two independent large, non-interventional, prospective, observational phase 4 single-arm studies have been initiated in Germany. Fingolimod is being assessed in the 5-year Post-authorization Non-interventional German Safety Study of Gilenya^®^ in MS Patients (PANGAEA) [32]. BRACE therapies were assessed in the independent, recently completed 2-year Prospective Pharmacoeconomic Cohort Evaluation (PEARL) [33]. PANGAEA and PEARL were conducted in Germany to allow analyses of prospectively collected data for substantial cohorts of patients in a single healthcare system that provides a wide range of treatment options for MS [34]. Germany has a relatively high prevalence of MS, with an estimated 130 000 people being affected [35, 36]; this permitted enrolment of a large number of patients. Owing to the fact that patients were recruited from the same country and centers and during an overlapping time period, environmental and pharmacoeconomic conditions are likely to have been similar.

The aim of this study was to evaluate real-world clinical outcomes in propensity-score-matched patients with active MS receiving either fingolimod or BRACE therapies using data from the two independent, single-arm registry studies, PANGAEA and PEARL. To compare data from separate, non-interventional studies, a novel methodological approach was developed and used.

## Methods

### PANGAEA and PEARL study designs

Patients with RRMS receiving fingolimod 0.5 mg were recruited into PANGAEA between April 2011 (when market approval was received for fingolimod in the EU) and December 2013, with the planned observational period ending in December 2018 [32]. Patients with RRMS receiving BRACE therapies for at least 30 days were recruited into PEARL between September 2010 and March 2011, with the observational period ending in March 2013 [33]. In both studies, prospective visits were conducted at intervals of 3 months.

### Patient selection and propensity score matching

Patients with RRMS from the PANGAEA and PEARL studies were included in this analysis if they met all the inclusion criteria, including providing informed consent, receiving BRACE therapies before participating in the study, having at least one relapse in the year before the study, and not having missing information on the number of relapses in the year before recruitment. PANGAEA patients who had previously participated in PEARL were excluded. To compare clinical outcomes between the independent PANGAEA and PEARL single-arm registry studies, a novel methodology was developed. Patients in the PANGAEA cohort were matched in a 3:1 ratio to patients in the PEARL cohort using a propensity-score-matching approach. All patients were included in the propensity-score analysis regardless of whether they dropped out at a later stage in the study. Propensity scores were calculated as the probability of enrolment in PANGAEA (vs PEARL) according to the following baseline predictors: number of relapses in the preceding year; time since diagnosis of MS; previous type of BRACE therapy (Betaseron, Rebif, Avonex, Copaxone and Extavia); Expanded Disability Status Scale (EDSS) score; age; and sex. Full details of the propensity score model are presented in Supporting Information (S1 Supporting information). Subgroups of matched patients in the PANGAEA and PEARL cohorts were identified according to the additional criterion of having at least 1 year of follow-up.

### Study outcomes

Relapses, 3-month and 6-month confirmed disability progression (CDP), and 3-month and 6-month confirmed disability improvement were assessed using follow-up data from the PANGAEA and PEARL study records. Relapses were not defined according to any pre-specified criteria but were assessed according to the clinical judgement of physicians in the real world. CDP was assessed according to increases in EDSS score, with confirmation of the increase in disability at a visit in the absence of a relapse as follows: a 1.5-point increase from baseline in patients with a baseline EDSS score of 0; a 1-point increase from baseline in patients with baseline EDSS scores of 1–5.0; and a 0.5-point increase in patients with baseline EDSS scores of 5.5 or more. A 3-month CDP required that the initial EDSS score at onset of disability progression, the 3-month confirming EDSS score and all EDSS evaluations in between met the disability progression criteria. Time to 3-month CDP was calculated using a threshold of 76 days (the protocol allows a visit window of ± 14 days for any 3-month interval visits) as the lower end of the 3-month visit and the corresponding threshold for 6-month CDP was 166 days. Confirmed disability improvement was defined as a decrease in EDSS score of at least 1 point for all patients regardless of baseline EDSS score, with the decrease in disability confirmed in a similar manner to that used for CDP. The number of relapses was assessed according to relapse dates. Relapses separated by fewer than 28 days were counted as a single relapse.

At each study visit, patients also reported the number of days of sick leave during the preceding 3 months. Days of sick leave were averaged over all visits during a patient’s participation in the study. Patient disease severity was assessed by clinicians using the Clinical Global Impressions (CGI) scale [37], which was administered at baseline and at months 12, 18 and 24. The CGI scale requires clinicians to rate the severity of a patient’s illness, relative to the clinician’s experience of patients with the same diagnosis, on a scale from 1 (patient is not at all sick) to 7 (patient is extremely sick) [37].

### Statistical analyses

Annualized relapse rates (ARRs) were analyzed using a negative binomial regression model. The model used the number of relapses as the response variable and the logarithm of time on study as an offset variable. The ARR ratio (patients treated with fingolimod vs those receiving BRACE) and its associated 95% CI and *p*-value were estimated from this model. Times to 3-month and 6-month CDP, and 3-month and 6-month confirmed disability improvement were estimated using a Kaplan–Meier approach, and differences were assessed using a log-rank test. HRs for the relative probabilities of CDP and confirmed disability improvement (fingolimod vs BRACE) were estimated using a Cox proportional hazards model adjusted for treatment. Patients were assessed for outcomes of interest until the point of drop out, at which point they were censored. In analysis of rates, only the period over which patients were part of the study were considered. In each analysis of ‘time-to’ outcomes, patients without the outcome event were censored at their last available visit. The proportions of patients in each cohort who were free from both relapses and disability progression were compared using a Fisher’s exact test. Similar analyses were performed for the subgroup of matched patients with at least 1 year of follow-up. To allow for any residual confounding, analyses were also conducted with adjustment for baseline covariates (age and sex) and pre-study clinical characteristics (pre-study relapses, time since diagnosis, baseline EDSS score and pre-study DMT). For sick leave and CGI analyses, data were only available for a subset of patients, and were analyzed in the two matched cohorts using a two-sided Jonckheere–Terpstra test.

## Results

### Study population and cohort characteristics

In total, 2255 and 587 patients met the inclusion criteria in the PANGAEA and PEARL cohorts, respectively. Baseline demographic and clinical characteristics are shown in Table 1. On average, compared with patients in PEARL, those in PANGAEA had a diagnosis of MS for longer (mean duration, 7.7 vs 5.8 years), had a slightly higher EDSS score (mean, 2.9 vs 2.4) and were more likely to have had multiple relapses in the previous year (56.7% vs 30.1%). After propensity score matching, 1287 and 429 patients were retained for further analysis in the PANGAEA and PEARL cohorts, respectively. There were no clinically significant differences between the propensity-score-matched cohorts (Table 1 and Supporting Information [S1 Supporting information]). Mean follow-up time was slightly shorter in the PANGAEA cohort than in the PEARL cohort (for both the matched and unmatched populations); however, total study time in patient-years was longer in the PANGAEA cohort than the PEARL cohort (for both the matched and unmatched populations) owing to differences in the size of the cohorts. A subgroup of matched patients with the additional criterion of having at least 1 year of follow-up data included 730 patients in PANGAEA and 325 in PEARL (S2 Table). The proportion of patients discontinuing the study during the follow-up period was comparable between the PANGAEA and PEARL cohorts in the unmatched (19.8% and 18.2%, respectively) and matched population (18.6% and 20.3%, respectively).

**Table 1:** Baseline demographic and clinical characteristics in the unmatched and propensity-score-matched PANGAEA and PEARL cohorts.

Characteristic	Unmatched		Propensity score-matched	
	PANGAEA (fingolimod, n = 2255)	PEARL (BRACE, n = 587)	PANGAEA (fingolimod, n = 1287)	PEARL (BRACE, n = 429)
Age, years				
Mean (SD)	38.8 (10.1)	39.8 (10.2)	39.2 (10.0)	39.3 (10.1)
Median (range)	39 (17–85)	41 (19–66)	40 (17–69)	40 (19–66)
Sex, n (%) female	1618 (71.8)	444 (75.8)^a^	951 (73.9)	309 (72.0)
Number of relapses in previous year, n (%)				
1	977 (43.3)	410 (69.8)	751 (58.4)	257 (59.9)
2	843 (37.4)	134 (22.8)	403 (31.3)	129 (30.1)
≥ 3	435 (19.3)	43 (7.3)	133 (10.3)	43 (10.0)
Time since MS diagnosis, years^b^				
Mean (SD)	7.7 (6.4)	5.8 (5.9)	7.0 (5.7)	6.8 (6.3)
Median (range)	6 (0–49)	4 (0–33)	6 (0–37)	5 (0–33)
EDSS score^b^				
Mean (SD)	2.9 (1.6)	2.4 (1.5)	2.7 (1.5)	2.7 (1.6)
Median (range)	2.5 (0–8.0)	2.0 (0–8.5)	2.5 (0–8.0)	2.5 (0–8.5)
BRACE therapy at baseline, n (%)				
IFN beta-1a	1051 (46.6)	276 (47.0)	613 (47.6)	204 (47.6)
IFN beta-1b	485 (21.5)	142 (24.2)	294 (22.8)	92 (21.4)
Glatiramer acetate	719 (31.9)	169 (28.8)	380 (29.5)	133 (31.0)
Time on study, years				
Mean (SD)	1.2 (0.8)	1.6 (0.6)	1.3 (0.8)	1.5 (0.6)
Total (patient-years)	2793.5	931.6	1673.0	655.6

^a^For one patient, information was missing on sex. The proportion of female patients was therefore calculated based on a total population of 586 patients.

^b^Not all patients in the unmatched cohorts had sufficient data to be included in these analyses.

BRACE, Betaseron^®^, Rebif^®^, Avonex^®^, Copaxone^®^, Extavia^®^ (beta interferons or glatiramer acetate); EDSS, Expanded Disability Status Scale; IFN, interferon; MS, multiple sclerosis; PANGAEA, Post-authorization Non-interventional German Safety Study of Gilenya^®^ in Multiple Sclerosis Patients; PEARL, Prospective Pharmacoeconomic Cohort Evaluation; SD, standard deviation.

### Relapse rates in the PANGAEA and PEARL cohorts

**Fig 1:**
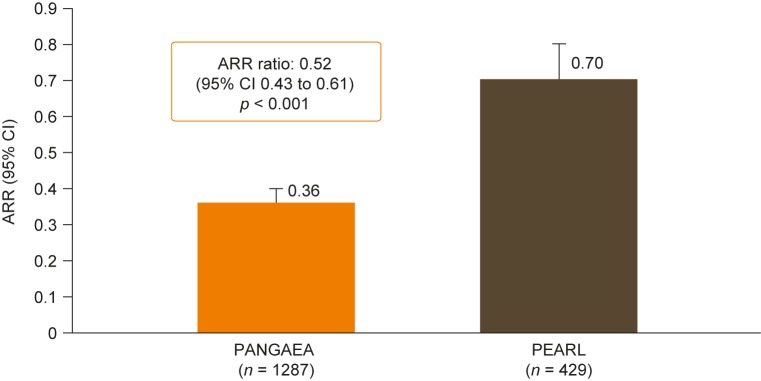
Unadjusted annualized relapse rates. Unadjusted ARRs in propensity-score-matched patients from the PANGAEA (fingolimod) and PEARL (BRACE) cohorts. Unadjusted ARR ratios were calculated from a negative binomial regression model using PEARL as the reference cohort. ARR, annualized relapse rate; BRACE, Betaseron^®^, Rebif^®^, Avonex^®^, Copaxone^®^, Extavia^®^ (beta interferons or glatiramer acetate); CI, confidence interval; PANGAEA, Post-authorization Non-interventional German Safety Study of Gilenya^®^ in Multiple Sclerosis Patients; PEARL, Prospective Pharmacoeconomic Cohort Evaluation.

**Fig 2:**
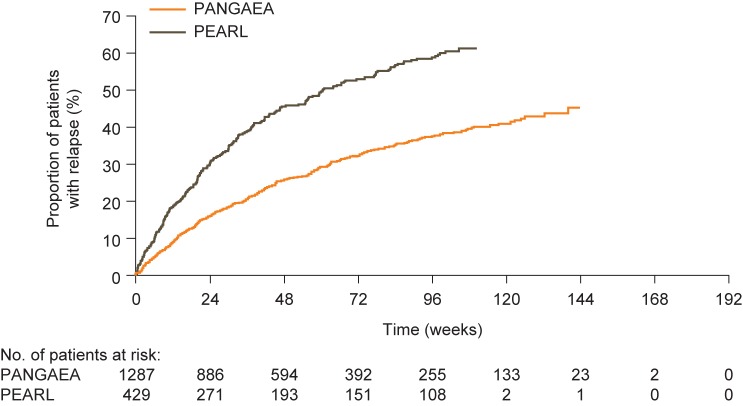
Time to first relapse in propensity-score-matched patients from the PANGAEA (fingolimod) and PEARL (BRACE) cohorts. Kaplan–Meier estimate (cumulative distribution function) showing time to first relapse in propensity-score-matched patients from the PANGAEA (fingolimod) and PEARL (BRACE) cohorts. BRACE, Betaseron^®^, Rebif^®^, Avonex^®^, Copaxone^®^, Extavia^®^ (beta interferons or glatiramer acetate); PANGAEA, Post-authorization Non-interventional German Safety Study of Gilenya^®^ in Multiple Sclerosis Patients; PEARL, Prospective Pharmacoeconomic Cohort Evaluation.

In the overall unmatched PANGAEA and PEARL cohorts, ARRs were 0.41 and 0.62, respectively (*p* < 0.001). This trend was repeated in the propensity-score-matched cohorts, with the ARR being lower for patients in PANGAEA than for those in PEARL (0.36 vs 0.70; Fig 1). On average, propensity-score-matched patients in the PANGAEA cohort experienced 48% fewer relapses per year than those in the PEARL cohort (ARR ratio: 0.52; 95% CI: 0.43–0.61; *p* < 0.001; Fig 1). Similar results were observed for ARRs in the subgroup of propensity-score-matched patients with at least 1 year of follow-up (ARR ratio: 0.52; 95% CI: 0.43–0.64; *p* < 0.001) and in the propensity-score-matched cohorts adjusted for baseline covariates not included in propensity score matching (ARR ratio: 0.49; 95% CI: 0.42–0.58; *p* < 0.001). The time to first relapse was typically longer for patients in PANGAEA than for those in PEARL in propensity-score-matched analyses (Fig 2).

### Confirmed disability progression in the PANGAEA and PEARL cohorts

**Fig 3:**
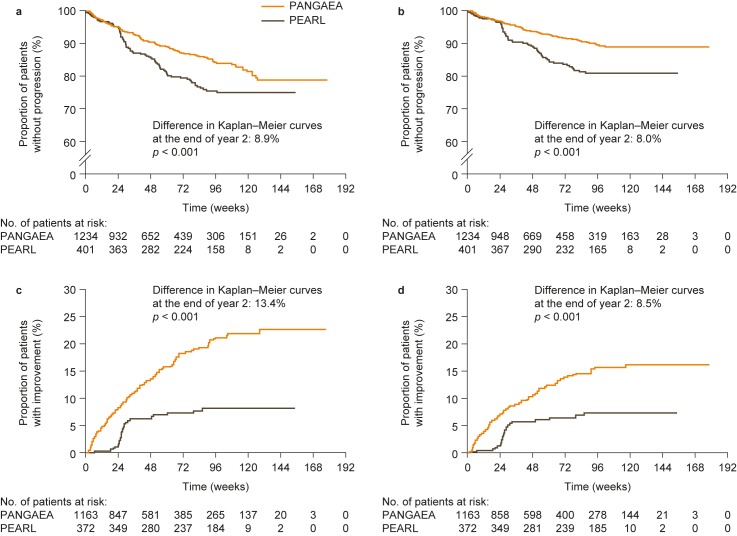
**Time to (a) 3-month and (b) 6-month confirmed disability progression, and (c) 3-month and (d) 6-month confirmed disability improvement in propensity-score-matched patients from the PANGAEA (fingolimod) and PEARL (BRACE) cohorts.** Kaplan–Meier estimate (cumulative distribution function) showing time to cumulative disability progression and time to cumulative disability improvement in propensity-score-matched patients from the PANGAEA (fingolimod) and PEARL (BRACE) cohorts. Not all matched patients had available Expanded Disability Status Scale scores and therefore some individuals could not be included in these analyses. BRACE, Betaseron^®^, Rebif^®^, Avonex^®^, Copaxone^®^, Extavia^®^ (beta interferons or glatiramer acetate); PANGAEA, Post-authorization Non-interventional German Safety Study of Gilenya^®^ in Multiple Sclerosis Patients; PEARL, Prospective Pharmacoeconomic Cohort Evaluation.

In the overall unmatched PANGAEA and PEARL cohorts, Kaplan–Meier estimates for the proportions of patients with 3-month CDP at the end of year 2 were 16.4% and 22.0%, respectively (log-rank test *p* = 0.008); proportions of patients with 6-month CDP were 11.1% and 19.1%, respectively (*p* < 0.001). In propensity-score-matched cohorts, the risk of 3-month CDP was significantly reduced in the PANGAEA cohort compared with the PEARL cohort (37% reduction; HR, 0.63; 95% CI: 0.48–0.83; *p* < 0.001; Fig 3A). The risk of 6-month CDP in the matched cohorts was also significantly reduced in PANGAEA compared with PEARL (47% reduction; HR, 0.53; 95% CI: 0.39–0.74; *p* < 0.001; Fig 3B). In the subgroup of propensity-score-matched patients with at least 1 year of follow-up, similar reductions were observed in the risk of both 3-month CDP (30% reduction; HR, 0.70; 95% CI: 0.52–0.94; *p* = 0.019) and 6-month CDP (39% reduction; HR, 0.61; 95% CI: 0.44–0.85; *p* = 0.004). Comparable results for the risk of both 3-month CDP (38% reduction; HR, 0.62; 95% CI: 0.47–0.82; *p* < 0.001) and 6-month CDP (47% reduction; HR, 0.53; 95% CI: 0.38–0.73; *p* < 0.001) were observed in the matched cohorts when analyses were adjusted for baseline covariates.

Among patients in the unmatched cohorts for whom data were available (PANGAEA, n = 2009; PEARL, n = 548), a significantly higher proportion in PANGAEA than in PEARL were free from both relapses and 3-month (65.3% vs 42.3%; *p* < 0.001) or 6-month CDP (67.4% vs 42.9%; *p* < 0.001). Similar results were seen in the propensity-score-matched PANGAEA and PEARL cohorts for the proportions of patients who were relapse-free and CDP-free (PANGAEA, n = 1234; PEARL, n = 401; relapse-free and 3-month CDP-free: 65.7% vs 38.7%; *p* < 0.001; relapse-free and 6-month CDP-free: 68.2% vs 39.2%; *p* < 0.001). Results were also consistent in the subgroup of propensity-score-matched patients in the PANGAEA and PEARL cohorts with at least 1 year of follow-up (PANGAEA, n = 720; PEARL, n = 325) for the proportions of patients who were free from relapses and 3-month (57.9% vs 39.1%; *p* < 0.001) or 6-month CDP (60.8% vs 39.4%; *p* < 0.001).

### Confirmed disability improvement in the PANGAEA and PEARL cohorts

The proportions of patients in the overall unmatched PANGAEA and PEARL populations (PANGAEA, n = 1924; PEARL, n = 499) with 3-month confirmed disability improvement at the end of year 2 were 15.2% and 6.6%, respectively (log-rank test *p* < 0.001). Similar results were seen for 6-month confirmed disability improvement (11.5% vs 5.8%; *p* < 0.001). In the propensity-score-matched cohorts (PANGAEA, n = 1163; PEARL, n = 372), the probability of 3-month confirmed disability improvement was again significantly higher in PANGAEA than in PEARL (175% increase; HR, 2.75; 95% CI: 1.82–4.15; *p* < 0.001; Fig 3C). The probability of 6-month confirmed disability improvement was also significantly higher in PANGAEA than in PEARL for these matched cohorts (126% increase; HR, 2.26; 95% CI: 1.45–3.53; *p* < 0.001; Fig 3D). In the subgroup of propensity-score-matched patients with at least 1 year of follow-up (PANGAEA, n = 684; PEARL, n = 297), similar increases were observed in the probability of both 3-month (176% increase; HR, 2.76; 95% CI: 1.80–4.22; *p* < 0.001) and 6-month (136% increase; HR, 2.36; 95% CI: 1.50–3.69; *p* < 0.001) confirmed disability improvement. Adjustment for baseline covariates in the matched cohorts had little impact on analyses, with the probability of 3-month (168% increase; HR, 2.68; 95% CI: 1.75–4.12; *p* < 0.001) and 6-month (127% increase; HR, 2.27; 95% CI: 1.45–3.57; *p* < 0.001) confirmed disability improvement remaining significantly higher in PANGAEA than in PEARL.

### Physician-/patient-reported outcomes in the PANGAEA and PEARL cohorts

**Fig 4:**
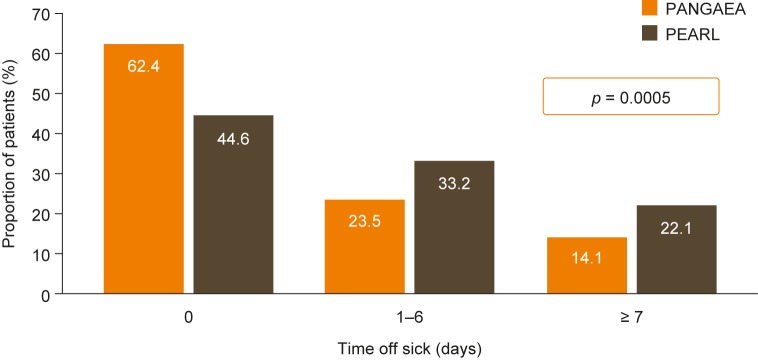
Proportions of patients with different numbers of days off work sick in 3 months. The proportions of patients were calculated in the propensity-score-matched PANGAEA (n = 149) and PEARL (n = 307) cohorts. PANGAEA, Post-authorization Non-interventional German Safety Study of Gilenya^®^ in Multiple Sclerosis Patients; PEARL, Prospective Pharmacoeconomic Cohort Evaluation.

Sick leave data were available for only a subset of the propensity-score-matched cohorts (PANGAEA, n = 149; PEARL, n = 307). The mean number of days off work sick during the preceding 3 months was lower in PANGAEA than in PEARL (4.2 vs 7.7 days), and patients in the PANGAEA cohort were significantly less likely than those in the PEARL cohort to have taken any sick leave (proportion with 0 days off sick: PANGAEA, 62.4%; PEARL, 44.6%; *p* = 0.0005; Fig 4). In the matched cohorts with CGI data (PANGAEA, n = 1207; PEARL, n = 427), analysis of the change in CGI score from baseline to the last available visit showed a higher proportion of patients with improvement in PANGAEA, and a smaller proportion with worsening status, than in PEARL (improvement: 28.2% vs 15.2%; worsening: 16.4% vs 30.4%; *p* < 0.0001). Similar results were seen for patients with 2 years of CGI data (PANGAEA, n = 389; PEARL, n = 249; improvement: 28.8% vs 16.1%; worsening: 17.5% vs 27.7%; *p* < 0.0001).

## Discussion

This long-term, real-world, observational study developed and used a novel methodological approach to compare important outcomes of disease among patients with active MS from two separate, single-arm, prospective, observational studies: PANGAEA, in which patients received fingolimod, and PEARL, in which they received BRACE therapy. Treatments for MS aim to reduce disease activity [8], but in some patients this remains high despite BRACE therapy, prompting a switch to an alternative treatment [38–40]. The results of the present study focus on patients with active disease who are receiving BRACE therapy, the indication for which fingolimod is approved in the EU [19]. This study found that in clinical practice, switching to fingolimod treatment is more effective than continuing BRACE therapy in improving a number of clinical and physician-/patient-reported outcomes, including relapse rates, disability progression and improvement, and measures of productivity in patients with active MS.

In our propensity-score-matched cohorts, we observed a 48% reduction in the number of relapses in patients receiving fingolimod in PANGAEA compared with those continuing to be treated with BRACE in PEARL. The relative reduction in ARR observed in PANGAEA and PEARL was consistent with the findings of a *post hoc* analysis of data from the phase 3 TRANSFORMS [27]. In patients with high disease activity despite receiving a DMT in the year before entering TRANSFORMS, fingolimod reduced the ARR by 50% versus IFN beta [27]. In addition, the observed relative reduction in relapses in our study is consistent with that seen in recent real-world evidence studies [29, 30, 41]. In a US administrative claims database study, a 50% reduction in ARR was found in patients with a history of relapse treated with fingolimod, compared with those receiving BRACE therapies [30]. It is notable that similar ARR reductions were seen despite differences in baseline demographic and clinical characteristics between the US database study and the present analysis [30]. An additional claims database study found a 62% reduction in ARR in patients switching therapy from IFN beta to fingolimod compared with those switching to glatiramer acetate [29]. The observations in PANGAEA and PEARL are also similar to those of a global MSBase registry study [31]. Patients switching from BRACE to fingolimod had a lower mean ARR (0.31 vs 0.42) and a 26% decrease in hazard of first on-treatment relapse compared with those switching from one BRACE therapy to another [31]. On average, patients in the MSBase analysis had a similar duration and severity of disease to those in the present study [31]. In summary, although the magnitude of ARR reduction varies among studies, reductions in relapse rates for fingolimod compared with BRACE are seen both in the present study and across phase 3 trial data and real-world evidence studies, despite differences in patient baseline characteristics and study types [27, 29–31, 41].

In our study, there was a 37% reduced risk of 3-month CDP and a 47% reduced risk of 6-month CDP in the PANGAEA cohort versus the PEARL cohort. Furthermore, patients in PANGAEA were more likely to be free from both relapses and CDP than patients in PEARL. A recent analysis of the global MSBase registry has identified a similar reduction in the risk of CDP [42]. Patients whose therapy was switched from BRACE to fingolimod in the MSBase study had a 47% reduced risk of 3-month CDP compared with those switching from one BRACE therapy to another [31]. The results of the present study, conducted in a single healthcare system using data collected prospectively in two independent studies according to a predefined protocol, confirm the reduction in risk of CDP with fingolimod in a larger sample of patients than that used in the MSBase study. In addition to the observed reduction in the risk of CDP, the relative probability of experiencing 3-month and 6-month confirmed disability improvement was increased by 175% and 126%, respectively, for PANGAEA versus PEARL. These findings are consistent with those of *post hoc* analyses of data from FREEDOMS and TRANSFORMS, which show that fingolimod may be associated with confirmed improvement in disability [42].

Relapses and disability progression are associated with significant reductions in quality of life, and can lead to loss of employment [43–45]. Advanced states of disability and relapses are associated with increased use of healthcare resources, and increased direct costs and indirect costs, such as costs associated with sick leave and informal care [44–46]. In our analysis, patients with active disease who switched to fingolimod in PANGAEA were less likely than those who continued on BRACE therapy in PEARL to have taken time off work in the preceding 3 months, and were more likely to have improved CGI scores. By reducing the rate of relapses and disability progression, fingolimod may improve patients’ quality of life and productivity, and reduce the burden of MS [47]. Many patients with MS who experience relapses despite receiving a previous DMT would benefit from a therapy with a drug with these qualities; our findings therefore have clinical relevance. Other treatment options for this group include natalizumab. In real-world studies comparing fingolimod and natalizumab, results are inconclusive regarding the comparative effectiveness of these treatments: some studies report that natalizumab and fingolimod have similar effectiveness [48, 49] and other studies suggest that natalizumab is more effective than fingolimod.[50–52] Reasons for differences in trends across studies may be attributed to differences in study design, patient characteristics or data sources across the publications.

A strength of the PANGAEA and PEARL registries is that they contain high-quality data owing to the use of external validation checks at the time of, and following, data entry. More generally, prospective, observational trials allow for the assessment of long-term, real-world clinical outcomes in patient populations that can be regarded as being more representative of clinical practice than those enrolled in randomized controlled trials [28]. The use of this methodology, particularly when supported by specific software tools such as the MS management system, MSDS 3D [53], is effective in terms of both time and cost because the data assessed are collected from visits scheduled as part of local clinical practice [54, 55]. As part of this study, a novel methodology was developed to compare treatments between propensity-score-matched cohorts of patients from separate, single-arm, non-interventional studies. This approach to generating high-quality real-world data from independent studies probably has more general applications for measuring treatment benefit in clinical practice, beyond the field of MS, and also expands the data sources that can be used for real-world evidence generation.

One of the general limitations of phase 4 observational studies is the difficulty in determining whether patients have used DMTs as prescribed. Therefore, DMT exposure is presumed and, in the case of injectable DMTs, which have been shown to be associated with lower persistence and adherence than oral DMTs [56], may be overestimated. The results of the analyses in the present study are, however, consistent with the results of the fingolimod phase 3 trials. Another limitation of observational studies is the lack of randomization and blinding, which can lead to confounding and bias between the patient cohorts. To compensate for differences between study cohorts in the present study, we used propensity score matching to select comparable patient cohorts for this analysis. While some caution is needed in the interpretation of results arising from this type of statistical approach, propensity score matching is a relatively robust technique to reduce the chances of differences in outcomes being attributable to measured covariates. Nevertheless, propensity score matching cannot account for other possible important but unknown or unmeasured covariates, such as MRI lesion activity. Fingolimod treatment resulted in more favorable relapse and CDP outcomes, both in propensity-score-matched cohorts and in additional analyses adjusted for residual confounding. It should be noted that both PANGAEA and PEARL were conducted only in Germany, which allows a better comparison of the data than if the studies had been conducted in different countries. At present, patients with MS in the German healthcare system have a wide range of available treatment options [34, 57], and assignment to receive fingolimod or BRACE therapies is unlikely to be confounded by potential treatment access issues. The results for these observational study populations are likely to be broadly applicable to the overall MS population in Germany; however, caution may be needed in generalizing the results of these studies to other settings in which patients may have had different treatment in the earlier stages of MS.

The results of this study suggest that in the real world, long-term fingolimod treatment is more effective than injectable BRACE therapies in patients with MS with disease activity despite previous DMT use. This study also developed a novel methodology to compare data from two separate, single-arm, prospective, non-interventional studies, which is likely to have more general applications for generating data for other real-world studies.

## Supporting information

S1 Supporting information Propensity-score matching. (PDF)

S1 Table Inclusion and exclusion criteria for propensity score matching. (PDF)

S2 Table Study populations. (PDF)

S3 Table Multivariable logistic regression of the probability of fingolimod treatment (vs BRACE). (PDF)

S1 Fig Distribution of propensity scores before and after matching. (EPS)
