# Supplementary material for: Two studies in one: A propensity-score-matched comparison of fingolimod versus interferons and glatiramer acetate using real-world data from the independent German studies, PANGAEA and PEARL
Source: PLoS One. 2017 May 5;12(5):e0173353. doi: 10.1371/journal.pone.0173353 (PMC5419529; doi:10.1371/journal.pone.0173353)
Supplement: S1 Supporting information — (PDF) [file pone.0173353.s001.pdf]

S1 Supporting information. Propensity score matching

In the absence of randomization, exposure to particular therapies (i.e. treatment assignment) is likely to be influenced to some degree by baseline characteristics that are independently related to disease outcomes. The interpretation of treatment effects is therefore complicated by the presence of potential confounding factors. One approach commonly used to control for confounding in studies involving two treatments (or one treatment and a control) is propensity score matching. Propensity score matching uses a model of the probability of receiving a treatment as a function of potentially predictive factors. By matching the propensity scores of individuals in the two treatment groups, cohorts of matched patients are generated. After matching, differences in outcomes between the two cohorts are not attributable to the measured covariates. Unlike randomization, however, propensity score matching cannot adjust for the presence of unknown confounding factors.

In our analysis, patients were considered for propensity score matching based on the inclusion and exclusion criteria in S1 Table. In total, 2842 patients were included, comprising 2255 in PANGAEA (Post-authorization Non-interventional German Safety Study of Gilenya® in Multiple Sclerosis Patients) and 587 in PEARL (Prospective Pharmacoeconomic Cohort Evaluation; S2 Table). Logistic regression was used to estimate propensity score. The dependent variable for the propensity score model was the treatment arm (fingolimod [PANGAEA] vs BRACE [PEARL]). The model therefore estimates the probability of receiving fingolimod treatment for each patient. Possible predictive baseline/pre-study factors included in the model were: age at baseline (years); sex; time since diagnosis at baseline (years); treatment at baseline (BRACE); number of relapses during the 12 months before baseline (categorical: 1, 2, ≥ 3); and Expanded Disability Status Scale score at baseline. In total, 384 patients had missing data for at least one of these factors and were excluded from

the analysis. The propensity score was matched using the nearest-neighbor method using macro *PSMatch_Multi.sas* (available from http://www.lexjansen.com/nesug/nesug10/ad/ad05.pdf) with a tolerance of 0.01. A 3:1 matching ratio was used owing to the available number of patients in each study.

The propensity-score-matched cohorts comprised 1287 patients from PANGAEA and 429 patients from PEARL (S2 Table). Estimates of the multivariable logistic regression parameters in the model are shown in S3 Table. The distribution of propensity scores before and after matching is shown in S1 Fig..
