# Supplementary material for: Two studies in one: A propensity-score-matched comparison of fingolimod versus interferons and glatiramer acetate using real-world data from the independent German studies, PANGAEA and PEARL
Source: PLoS One. 2017 May 5;12(5):e0173353. doi: 10.1371/journal.pone.0173353 (PMC5419529; doi:10.1371/journal.pone.0173353)
Supplement: S1 Table — (PDF) [file pone.0173353.s002.pdf]

S1 Table. Inclusion and exclusion criteria for propensity score matching.

Inclusion criteria	Exclusion criteria
• Patient consent has been given<br>• Patient has been categorized with disease type 'RRMS'<br>• Patient has received BRACE before participating in the study<br>• Patient has had at least one relapse during the 12 months before the study	• PANGAEA patients who participated in PEARL<br>• Patients who received no or non-BRACE treatment before the study<br>• Patients with no relapses during the 12 months before the study<br>• Patients with missing information for the number of relapses in the preceding 12 months

BRACE, Betaseron®, Rebif®, Avonex®, Copaxone®, Extavia®; PANGAEA, Post-authorization Non-interventional German Safety Study of Gilenya® in Multiple Sclerosis Patients; PEARL, Prospective Pharmacoeconomic Cohort Evaluation; RRMS, relapsing–remitting multiple sclerosis.
