# Supplementary material for: Two studies in one: A propensity-score-matched comparison of fingolimod versus interferons and glatiramer acetate using real-world data from the independent German studies, PANGAEA and PEARL
Source: PLoS One. 2017 May 5;12(5):e0173353. doi: 10.1371/journal.pone.0173353 (PMC5419529; doi:10.1371/journal.pone.0173353)
Supplement: S2 Table — (PDF) [file pone.0173353.s003.pdf]

S1 Table. Study populations.

Population, *n*	PANGAEA	PEARL	Total
**Analysis set**			
Total number of patients	4245	1784	6029
Inclusion criteria not fulfilled	1990	1197	3187
Inclusion criteria fulfilled	2255	587	2842
**Propensity score matching**			
Patients with missing data for predictive factors	378	6	384
Included in propensity score model	1877	581	2458
Not matched	590	152	742
Matched	1287	429	1716
Matched patients with at least 1 year of follow-up data	730	325	1055
**Physician-/patient-reported outcomes**			
Matched patients with sick leave data	149	307	456
Matched patients with Clinical Global Impressions scale data	1207	427	1634

PANGAEA, Post-authorization Non-interventional German Safety Study of Gilenya® in Multiple Sclerosis Patients; PEARL, Prospective Pharmacoeconomic Cohort Evaluation.
