# Supplementary material for: Two studies in one: A propensity-score-matched comparison of fingolimod versus interferons and glatiramer acetate using real-world data from the independent German studies, PANGAEA and PEARL
Source: PLoS One. 2017 May 5;12(5):e0173353. doi: 10.1371/journal.pone.0173353 (PMC5419529; doi:10.1371/journal.pone.0173353)
Supplement: S3 Table — (PDF) [file pone.0173353.s004.pdf]

S1 Table. Multivariable logistic regression of the probability of fingolimod treatment (vs BRACE).

**Parameter**	**Estimate**	**Standard error**	***p*-value**
Intercept	0.8101	0.2323	0.0005
Number of relapses in the 12 months before baseline[a]			
2	0.9360	0.1158	< 0.0001
≥ 3	1.4417	0.1765	< 0.0001
Number of years at baseline since diagnosis	0.0666	0.0101	< 0.0001
Baseline treatment[b]			
Avonex®	0.0114	0.1391	0.9347
Betaferon®	–0.1121	0.1591	0.4810
Extavia®	–0.3887	0.1957	0.0470
Rebif®	–0.1847	0.1378	0.1804
Baseline EDSS score	0.1686	0.0369	< 0.0001
Age at baseline, years	–0.0251	0.00564	< 0.0001
Male sex[c]	0.2556	0.1152	0.0265

[a]Reference = one relapse in the 12 months before baseline.

[b]Reference = Copaxone®.

[c]Reference = female.

BRACE, Betaseron®, Rebif®, Avonex®, Copaxone®, Extavia® (beta interferons or glatiramer acetate); EDSS, Expanded Disability Status Scale.
